# Badger Ecology, Bovine Tuberculosis, and Population Management: Lessons from the Island of Ireland

**DOI:** 10.1155/2024/8875146

**Published:** 2024-01-16

**Authors:** Andrew W. Byrne, Adrian Allen, Simone Ciuti, Eamonn Gormley, David J. Kelly, Nikki J. Marks, Nicola M. Marples, Fraser Menzies, Ian Montgomery, Chris Newman, Maria O'Hagan, Neil Reid, David M. Scantlebury, Peter Stuart, Ming-shan Tsai

**Affiliations:** ^1^One Health Scientific Support Unit, Department of Agriculture, Food and the Marine, National Disease Control Centre (NDCC), Agriculture House, Dublin 2, Ireland; ^2^Veterinary Science Division, Agri-Food and Biosciences Institute (AFBI), Stoney Road Stormont, Belfast BT43SD, Northern, Ireland; ^3^Laboratory of Wildlife Ecology and Behaviour, School of Biology and Environmental Science, University College Dublin, Dublin, Ireland; ^4^School of Veterinary Medicine, University College Dublin, Dublin, Ireland; ^5^Department of Zoology, School of Natural Sciences, Trinity College Dublin, Dublin, Ireland; ^6^School of Biological Sciences and Institute of Global Food Security (IGFS), Queen's University of Belfast, Northern Ireland, Belfast BT9 5DL, UK; ^7^Veterinary Epidemiology Unit, Department of Agriculture, Environment and Rural Affairs, Northern Ireland, Belfast, UK; ^8^Wildlife Conservation Research Unit, Department of Biology, The Recanati-Kaplan Centre, University of Oxford, Oxford OX13 5QL, UK; ^9^Department of Biological and Pharmaceutical Sciences, Munster Technological University, Clash V92 CX88 Tralee, Kerry, Ireland

## Abstract

The European badger, *Meles meles*, is an important wildlife host for *Mycobacterium bovis* and contributes to the epidemiology of bovine tuberculosis (bTB) in cattle in several countries. The control of zoonotic diseases, such as bTB, is a central component of global One-Health strategies. Such strategies are complicated by human–wildlife conflicts, particularly where wildlife reservoirs are legally protected. The contrasting objectives of disease management and wildlife conservation, therefore, can require significant investment in research to support evidence-based policies. In Britain and Ireland, for example, badgers are a legally protected species but are also subject to lethal control and vaccination for disease management. In this paper, we review recent (2012–2022) advances in research on this wildlife host on the island of Ireland, which is used to underpin national policies and identify research gaps. In recent years, significant advances in estimating key parameters related to badger management and population dynamics have been made, including estimating population abundance at varying scales (local, landscape, and national). Advances in tracking technology, integrated with mark-recapture and modelling tools, have provided significant insights into the movement ecology of badgers and their interactions with cattle. The adaptation of genetic technologies has improved our understanding of the transmission dynamics of *M. bovis* among different hosts. As a disease management strategy, the culling of badgers to control bTB has reduced badger densities significantly, although this is not considered a sustainable sole long-term solution to the problem of spillback infection. The recent development of vaccination strategies presents an additional approach to control the disease in wild populations. These types of interventions will require significant applied research to ensure they are sustainable and to maximise benefits. It is also expected that focused research efforts will improve human–wildlife coexistence in the context of the broader One-Health strategy.

## 1. Introduction

Medium-sized carnivores are an important, although sometimes overlooked, component of our biodiversity [1], often providing key ecosystem services (e.g., pest control, ecosystem engineering, etc.) and contributing to eco-epidemiological processes (e.g., hosts for parasitic or infectious pathogens) [2–6]. Where wildlife species are important hosts for zoonotic pathogens, there is a critical need to develop detailed insights into their ecology. Understanding their distribution, abundance, and factors that influence their population dynamics is necessary in order to support evidence-based policies and practical guidance for mitigation or disease management [7, 8]. Bovine tuberculosis (bTB), caused primarily by *Mycobacterium bovis*, is a globally distributed zoonotic disease primarily of cattle [9, 10]. However, the pathogen has been found in many wildlife host populations across the globe, including several deer species (e.g., North America; Europe), brush-tailed possums (New Zealand), buffalo (Australia), lions (South Africa), and wild boar (e.g., continental Europe), where spillback infection from wildlife to domestic hosts can be a significant challenge to bTB control and eradication programmes [9–11]. Badgers (*Meles meles*), a widespread and locally abundant mustelid found across Europe [12], have been implicated in the multispecies epidemiology of bTB in several countries in western Europe where bTB persists in cattle (e.g., France and Spain) [11]. However, in Great Britain (GB) and Ireland, where bTB has had a major impact on dairy and beef cattle production, badgers are an important reservoir of infection among wildlife hosts [8, 13].

As in several countries across the globe that experienced the economic impacts of bTB outbreaks [14], Britain and Ireland have been facing the twin challenges of biodiversity conservation and the control of zoonotic pathogens. Experiences on the island of Ireland demonstrate the challenge of bTB control in a domestic host when a wildlife reservoir is present. The research undertaken there is also an exemplar for the development of approaches to reduce badger welfare concerns, where feasible, while also attempting to reduce the impact of bTB on human economic activity and animal health. Such efforts in the past decade [15] have resulted in a significant body of research undertaken to fill some of the knowledge gaps required to accelerate the control of the disease. Here, we review recent research (2012–2022) from the state of Ireland (IE), also described as the Republic of Ireland, and Northern Ireland (NI), to identify the challenges and the means to address some of the constraints to eradication. We refer to the island as “Ireland,” which is assumed to be a single biogeographical and epidemiological unit, and the separate jurisdictions therein as IE and NI, respectively. We also reflect on research advances from elsewhere and the essential need for multidisciplinary research spanning farming and conservation interests, education and training, and cooperation among stakeholders to inform complex and controversial topics, particularly where management goals may conflict.

## 2. Fundamentals of Wildlife Disease Management

Wildlife disease management is predicated on understanding key variables about the target population and addressing several key questions—how large is the population, where is it distributed, how is the infection spread across this distribution, how connected is this population, and where are the infectious contacts likely to occur between domestic and wildlife populations [7, 11]?

### 2.1. Badger Population Estimation

#### 2.1.1. National Estimates—The Challenge of Scale

Estimating wildlife population parameters with accuracy at a large spatial scale is difficult and can make the management, monitoring, cost, and, ultimately, efficacy of disease-related interventions challenging [16, 17]. For badgers, population size estimates at large scales have relied on the identification and enumeration of setts (burrows) in the landscape [15]. Models make assumptions regarding the largest sett type, called “main setts,” as representing badger “clans” or social groups [15]. Once estimates of the number of social groups are made, they can be multiplied by a mean group size. Feore and Montgomery [18] demonstrated marked habitat effects on territory and group size using capture-mark-recapture methods (possibly associated with habitat carrying capacity or food availability [19]), indicating that adjustments of estimates are needed, depending on landscape composition. There have been no recent prospective surveys estimating badger social group numbers throughout the island of Ireland. In NI, data up to 2012 indicated a broadly stable estimate of 7,600 (6,200–9,000) social groups [20] based on a systematic survey. Using group size estimates from Feore and Montgomery [18] across three landscape types, these data suggested a total population of 34,100 (95% confidence interval (CI) 26,200–42,000) badgers. The estimates for IE are more uncertain, given the complex history of wildlife interventions [21], including the culling and vaccination of badgers as part of a bTB national control programme. Byrne et al. [22] used data from badger culling activities and a cross-validated species distribution model to estimate that the national population of IE was composed of approximately 19,000 (95% CI 12,000–28,000) social groups. However, simplistic multiplicative methods to estimate abundance may derive biased estimates, as it fails to account for any impact of population management interventions. As part of the badger bTB management programme, 30% of agricultural areas in IE have had some form of culling [23] or bTB vaccination [24], which impacts local abundance. A simulation model, incorporating the effects of landscape, culling history, and vaccination, estimated the badger population of IE at 63,000 (95% CI 48,000–79,500) individuals [21]. Modelling the cessation of culling in favour of vaccination increases the population estimation in IE to 92,000 (95% CI 67,000–119,000) individuals [21]. Taken together, these estimates suggest that the island of Ireland may have roughly 100,000 (95% CI 75,000–120,000) badgers across 25,000 (95% CI 18,000–37,000) social groups, with potential for increases in population size in the event of cessation of culling in IE.

Future studies using available technologies (e.g., non-invasive hair sampling, genetic fingerprinting, and camera trapping) are essential for obtaining greater precision in population estimates. Given the economic impact of bTB, its political importance, and the necessity to target and assess the efficiency of badger population interventions, there is a need for regular population monitoring and surveillance such that temporal variability in numbers may be predicted dynamically. This requires further work to establish spatial and temporal variation in social group sizes and factors impacting its variation. Climatic factors influence badger population growth [25], and therefore, the potential impacts of climate change on population size are also important for predicting future population trajectories.

#### 2.1.2. Local Estimates—The Tyranny of the Mean

Localised estimates of social group size, density, and abundance are more easily obtained with narrower CIs than national population estimates due to the reduced scale and focal intensity of such research.

In NI, the Department of Agriculture, Environment and Rural Affairs (DAERA) has conducted a 5-year “Test-and-Vaccinate-or-Remove” (TVR) Project within a 100 km^2^ study area in County Down [26]. Badger abundance was estimated annually using three mark-recapture methodologies: the Lincoln–Petersen method [27], the Chapman estimator [28], and a Bayesian estimation [29]. Results were averaged to provide a single estimate suggesting a density of 5.3–6.3 badgers/km^2^, which overlapped with, but was 50% higher than, central estimates for County Down produced from simple multiplicative sett survey models [20]. Some of this error may be due to the latter including the whole county and incorporating less optimal badger habitats. Spatiotemporal variations in badger density across the TVR Project area were also estimated as individual badgers were microchipped, and each trapping event was geolocated and recorded using a real-time data collection system [26]. This suggested only a very modest impact of removal on numbers, which did not differ significantly across the years, indicating a largely stable population.

Tessellations of estimated badger territories from active main sett locations using Thiessen polygons have been used to estimate variation in badger population size across a landscape of 755 km^2^ in County Kilkenny [30–32]. The overall density was estimated at approximately 1.1 badgers/km^2^ using a closed-subpopulation mark-recapture model [20]. However, this average did not reflect the significant local variation in density across the landscape. Using capture densities or minimum-number-alive (MNA), estimates suggest densities from <0.5 to > 10 badgers/km^2^ [30–32]. This significant density gradient was correlated with different movement metrics (see below), highlighting how differing carrying capacities can impact population dynamics but also potentially reflecting the *ghost* of previous management interventions. Another long-term project tracking badger movement in County Wicklow estimated badger population density using mark-recapture methods—an MNA estimator (1.2 badgers/km^2^) and the Chao method within the programme CAPTURE (1.8 badgers/km^2^) [33].

Current population estimates are based on invasive sampling using cull returns (trap-catch), live trapping, and mark-recapture, using estimators with known biases (e.g., MNA [34, 35]) or statistical models whose assumptions can be invalidated by the way badgers are trapped (e.g., low trapping efficacy, long inter-trap periods, non-contemporaneous trapping across study sites). Furthermore, badgers are difficult to capture due to their nocturnal habits, learned trap avoidance behaviour, and their response to weather conditions [36], resulting in highly variable trappability that also varies depending on the trapping method used (cage trapping versus wire restraint [20]). Capture is also associated with animal welfare issues such as capture-related injuries [37]. Greater use of non-invasive methods of population estimation, therefore, should be encouraged on both scientific and welfare grounds. Predicting changes in badger population size based on demographic models will require field data coupled with more detailed knowledge of badger reproduction and how this is affected by infection [38, 39].

### 2.2. Badger Movement Ecology

Badger movement ecology may be characterised at different spatial and temporal scales, providing insights into behaviour and space use by individuals or groups. Combining these approaches may help to understand the movement ecology of the species [40] and reveal the internal state (motivation) and external factors affecting movement. The movement patterns of badgers are also critical when considering their specific role in bTB epidemiology. The behaviour of individuals and the collective networks of interactions they form can have important implications for (intraspecific) maintenance of infection and (interspecific) spill-over/spillback infection to local cattle herds.

#### 2.2.1. Capture–Mark–Recapture Displacement—Snapshots in Time

Capture–mark–recapture has been used to estimate the “dispersal kernel,” or frequency distribution of movement step lengths (aka displacement) of badgers in agricultural landscapes in IE [41]. These displacements are measured using the straight-line (Euclidian) distance between capture locations. The frequency distribution clearly shows that badgers typically stay within a narrow range of movement distances of <1 km. Movement distances > 7 km are extremely rare. Such research suggests that rare long-distance movements by badgers could have been missed due to the limited spatial scale during previous studies [41]. This is consistent with the known territorial behaviour of badgers (see below), where individual badgers tend to roam across individual home ranges, which collectively form loose territories. The dispersal kernel, however, does little to explain why badgers undertake long-distance movements or what external factors may influence decisions to disperse.

Capture–mark–recapture models using data from County Kilkenny suggest that badger displacement is strongly influenced by the sex of the disperser, local density, and group composition (in terms of sex ratios and previous movement history [30–32]). Male badgers tend to move more frequently than females [30–32], but females tend to attempt more long-distance dispersals [41, 42]. This pattern is modulated by the composition of the social groups that these animals are moving from and to [31]. For example, males tend to move away from male-biased groups or are attracted to groups with more females. This suggests that both sexes maximise potential reproductive output or reduce within-sex competition. There was evidence of “vagrant” phenotypes, which tended to repeat movements, being associated with smaller social-group sizes [30, 32], a life history tactic also apparent in lower density populations in Spain [43]. The incidence of interterritorial movements is greater in lower density situations or where group sizes were inferred to be small [31]. Conversely, where group sizes are larger and densities higher, badger groups appear more stable with fewer recorded interterritorial movements. This suggests that social group dynamics may be affected by population density and the stability of the local population dynamics. However, this research from IE did not factor in the potential for the effects of historical culling or other disturbances.

Capture–mark–recapture data, in combination with genetic analysis, has been used to assess whether small-scale badger culling has any measurable effect on badger displacement in NI [44]. At these spatiotemporal scales, there was no evidence of any culling effect, at least where only small numbers of badgers were removed. However, it is well recognised that capture–mark–recapture methods cannot resolve the finer details of badger movement, which may be revealed using GPS tracking, dead-reckoning, and accelerometery. Field studies throughout the island of Ireland have started to generate data that describe badger behaviour in far greater detail than was previously known [42, 45–51].

#### 2.2.2. Intra- and Inter–specific Interactions

Proximity loggers are wearable technology that can record close contacts between tracked badgers to describe the contact rate within social groups and between individuals. O'Mahony [47] used such devices to study five badger social groups where 15 badgers were fitted with collars and 12,969 interactions were recorded. This study highlighted the importance of within-group dynamics where frequent and extended duration contacts occurred. Inter-group interactions were rare, comprising only 1% of all interactions recorded, highlighting the importance of measuring less frequent and potentially important events in longitudinal studies. The study also highlighted significant variations in individual behaviour as well as differences between males and females and among seasons (see below).

Such seasonally variable behaviours have also been described using camera trap-derived data. Caravaggi et al. [52] examined badger activity using 947 detections of badgers in several separate surveys of wild mammals. This showed that the onset of badger activity is less tied to sunset in summer and autumn than winter and spring, and unlike foxes (*Vulpes vulpes*) and pine martens (*Martes martes*), it was not correlated with the activity of potential prey species. This activity pattern complements the measurement of resting metabolic rate (RMR), which is an estimate of minimum energy requirements. McClune et al. [53] measured RMR in free-ranging badgers, demonstrating that RMR was greater than expected from allometric measurements in summer but not winter and autumn, suggesting a period of “winter lethargy” indicative of lower levels of activity and reduced body temperature. Barbour et al. [54] showed that bTB had no effect on RMR or daily energy requirement in badgers, suggesting that the costs of the disease are met by compensatory methods, given they are able to survive for long periods without adverse effects on energetics.

A study of badgers tracked using GPS collars in County Wicklow showed that they avoided cattle at pasture [45] and in farmyards [46]. This has been inferred to mean direct interspecific host-to-host transmission events are rare [55], despite a prevailing belief that aerosol-mediated transmission requiring close proximity (<1 m) is the predominant transmission mechanism [56]. Others highlight the idea that bTB may be transferred indirectly via fomites rather than directly between badgers and cattle [55]. Some studies have suggested that feeding sites and water sources provided for stock animals act as fomites [57], while the Wicklow studies suggest that pastures, as feeding locations, should also be considered as risk points. A field study in NI found that only 3% of agricultural fields in an area of moderate badger density (∼3 badgers/km^2^) had a badger sett and/or latrine present [58] (see also the discussion in Allen et al. [44]). Campbell et al. [58] also found a positive association between the time cattle spent in fields with badger setts, but not latrines or neighbouring cattle, and historical bTB herd breakdown risk.

O'Mahony [47], using camera trapping, found that badgers are the least frequent mammal visitors to farmyards in NI. Indirect interspecific contact can occur at badger setts and less commonly at water troughs and farm buildings, although the latter would appear to be much more infrequent than observed in Great Britain (GB) [59]. Camera traps have been used to investigate potential badger-cattle indirect interactions at likely fomites (places where pathogens might be present), suggesting that cattle have access to badger sett entrances while badgers have access to cattle water troughs [47, 55]. The observed frequency of cattle visits to badger-associated locations was more than three times greater than badger visits to cattle-associated locations, suggesting that physical interventions, e.g., fencing of setts to exclude cattle or the replacement of water troughs with cattle pasture pumps, may reduce indirect contact at some fomites [55]. Many biosecurity interventions are relatively inexpensive and within the skill sets of most farmers. While being recommended as part of biosecurity advice (e.g., [60, 61]), there have not been large-scale trials of such interventions to assess whether they would reduce cattle bTB breakdown risk.

#### 2.2.3. Tracking and Landscape Disturbance

GPS tracking was used to quantify the home ranges of badger groups during the TVR Project in County Down, NI. The home range area was not significantly altered by local badger removal [44, 51, 62], but badgers avoided crossing dual carriageways (divided highways), indicating that such structures impose a barrier to movement and reduce the probability of dispersal, at least at this study site [63]. Badger socio-spatial structure was also the focus of “the N11 badger study” [64] in County Wicklow. It investigated whether environmental disturbances, such as landscape change [65–67], in this case in the form of roadbuilding, can interfere with badger movement and territoriality in an area of up to 33 km^2^ [33]. Such disturbances may enhance the potential for bTB transmission [68] if behaviours change such that infectious contacts are increased between hosts. The N11 study was undertaken from April 2010 to October 2016, during which 80 badgers were collared, generating 81,925 location fixes. This large dataset allowed detailed observations of badger-ranging behaviour across several years. This facilitated investigation of the movement of adults when cubs were born [46], the activity of developing cubs/yearlings and their dispersal [65], the interaction of badgers with cattle [45, 46], the movement of adults from one social group to another involving both displacement and replacement of other adults [33], information gathering by adults of neighbouring social groups [48] and seasonal fluctuations in the ranging of resident badgers [49]. Road-building did not appear to affect movements (nightly distance, home range, and extraterritorial excursions) of badgers [49], suggesting that if fences were constructed to prevent badgers from gaining access to a road, or underpasses were constructed to allow badgers to cross new roads safely, badgers were unlikely to change their ranging behaviour in ways which would increase the potential for bTB spread.

While roadbuilding was shown to have a limited effect on badger ranges, the N11 study yielded insights into numerous behaviours. Super-ranging [33], a strategy where male badgers lay claim to more than one social group's territory, was a novel and surprising finding in an Irish context (see Revilla and Palomares [69] for similar observations in Spain). Some super-ranging males managed to maintain extended territories for up to 3 years, despite the obvious associated energetic demands. The project also identified extensive journeys made by dispersing female badgers as they sought new social groups [42]. Furthermore, badgers made extraterritorial excursions throughout the year [48]. These excursions were speculated to be for information gathering, i.e., monitoring neighbouring social groups, as well as opportunities for extragroup mating. All three of these ranging behaviours suggest that the territorial boundaries of badgers, at least within the medium-density social groups of County Wicklow [33], are more fluid than conventional ideas involving strong territoriality suggest.

These data [33], in combination with capture–mark–recapture data [30–32] and genetic evidence [70], suggest that non-rigid interpretation of badger socio-spatial organisation, across density gradients, are the norm. The relative strength of territoriality appears to increase with increasing density and carrying capacity, and decreases with population change [70, 71]. Badger populations may lie on different positions on the density-territorial gradient, leading to differing socio-spatial organisations. Where a given population sits within this gradient may predict the impact of demographic changes (e.g., via culling) or environmental disturbance (e.g., forest clearfelling or road building) on the dynamics of *M. bovis* transmission. However, the magnitude of the intervention or disturbance may also be important (e.g., selective culling versus proactive culling; clearfelling forests versus forest thinning). Epidemiological research has provided some evidence of an association between landscape disturbance caused by motorway building [72] or clearfelling of the forest [66, 67] and cattle herd bTB risk. However, it is not clear how these patterns emerged mechanistically via wildlife disturbance and how they fit within the density-territorial gradient framework (see below). In addition to the behavioural changes caused by disturbances increasing infectious contact, it is possible that stress could cause immunosuppression impacting the susceptibility to infection in wildlife [73]. George et al. [74] found that cortisol, a biomarker for physiological stress, was significantly higher in individuals testing positive for *M. bovis* in a badger population in NI.

#### 2.2.4. Dead-Reckoning and Accelerometry

The use of accelerometers to quantify animal behaviour and activity remotely was first suggested for use in diving seabirds in the late 1990s [75]. Significant advances, partially driven by the availability of inexpensive electronic sensors, have led to the development of tri-axial accelerometers and tri-axial magnetometers (generally driven by mobile phone technology), as well as GPS loggers [76]. One of the first studies to investigate behaviour in a terrestrial mammal was carried out with captive badgers and used simultaneous videos of behaviour to validate and predict behaviour from accelerometery [77]. Subsequently, McClune et al. [78] showed that accelerometery and relatively frequent (5 min) GPS-enabled dead-reckoning data could be combined to plot the track of a wild badger and elucidate its movements and relate these to habitat features, e.g., foraging in fields.

Magowan et al. [50] applied this GPS-enhanced dead-reckoning technology to track a population of badgers to show how different behaviours could be associated with certain areas and habitat features. It also highlighted that previous badger studies using GPS data, which are generally temporally well-spaced because acquiring positional fixes requires high power usage, are likely to have underestimated the distances travelled by animals and their precise space usage. This might be important in badgers as even brief visits to certain features, e.g., farm buildings, might increase the likelihood of interaction with livestock. In addition, because home range data determined using only GPS are calculated with fewer points, they may be far less representative compared with calculations of distributions of animal movements that use GPS-enhanced dead-reckoned data [79].

Badger movements, as revealed by GPS, appeared not to be affected by vaccination or selective culling [62], but the dead-reckoning data revealed that trapping badgers effects changes in behaviour [51]; also see Schutz et al. [80]. Assessment of the more intricate nuances of badger behaviour using acceleration data and determinations of GPS-enhanced dead-reckoning might reveal how such interference interacts with “perturbations” and may reveal where direct intra- and inter-specific pathogen transmission events are likely to occur. Accelerometry-based studies of badgers and other carnivores have also indicated that mass limits for attachment of tags should be increased due to forces much greater on animals during movement than the currently acceptable 3% of body weight [81].

### 2.3. Molecular Genetics Understanding the Past, Present, and Future

#### 2.3.1. Phylogeography and Genetic Population Structure

Developing a better understanding of the badger genetic population structure at differing spatial scales and the biotic and non-biotic forces that have shaped it across the island may aid disease control efforts. How wildlife population structure affects the partitioning of pathogen genetic diversity in the landscape could provide insight into disease transmission dynamics [82] and the role played by badgers [83].

Ireland has been an island for ∼15,000 years [84]. Colonisation by non-volant mammals after the Last Glacial Maximum 19–23 thousand years ago is an ongoing question for biologists seeking to explain Ireland's depauperate mammal fauna. There are ongoing debates about which species are native and when and how did others arrive, with the deliberate or inadvertent assistance of people [85–87]. Archaeological evidence suggests the badger as one of a group of economically important mammals introduced by Neolithic people for food, fur, or bone. O'Meara et al. [88] suggested Irish badgers exhibited an “Atlantic Fringe” genetic signature, with similarities to animals from the Iberian Peninsula, as has been noted for other species. Frantz et al. [89] expanded on this to suggest Irish badgers had a mixed heritage, with mitochondrial DNA (mtDNA) being most like contemporaries in Scandinavia but nuclear DNA being like badgers in GB. They hypothesised that badgers were transported to Ireland by humans, originally from GB, but subsequently, a larger incursion around the time of the Viking invasions founded most of the extant population. That study, however, relied on small numbers of badgers from a small number of locations. Allen et al. [90], using a survey of several hundred samples from across all of the islands of Ireland and Britain, identified two distinct Irish genetic clusters based on analysis of nuclear DNA. The largest and most geographically widespread of these clusters was found all over the island and was comprised of individuals possessing the Scandinavian-like mtDNA haplotypes. The second lineage was restricted to the east coast of the island and possessed a nuclear DNA signature similar to GB contemporaries. Badgers in these eastern areas also carried mtDNA haplotypes found commonly in Britain. Allen et al. [90] inferred that this east coast cluster likely arose from a small number of GB badgers translocated by human agency into Ireland, admixing with an already resident population with Scandinavian heritage. The median timing of introduction from GB was 600–700 years before the present–coincident with a period of human colonisation of Ireland from Britain, which occurred after the Viking invasions.

Landscape genetics unites landscape ecology and population genetics to provide insights into how landscape composition and configuration affect genetic processes such as gene flow, genetic drift, and selection [91]. Gene flow and its impediments can be useful alongside more conventional landscape ecology methods in providing information on habitat usage and connectivity [92]. Guerrero et al. [93] applied this interdisciplinary approach to badger genetic data collected across the island of Ireland [90]. They found that geographic distance and elevation were the major factors affecting gene flow on the island. Earthworm availability and landcover type did not affect genetic differentiation. This was broadly in line with the observation that badgers are generally philopatric, exhibiting significant isolation by distance at a variety of spatial scales [94], and in medium-density populations, such as that seen across Ireland, social group cohesion and territoriality are common [95]. However, a small proportion of badgers move over larger distances [41, 42], and the finding that there are no major physical barriers to badger dispersal could indicate the potential for infection to be spread among badger subpopulations.

#### 2.3.2. Wildlife Management

Studies in both Ireland and Britain have used population genetic indices and social group relatedness metrics to assess the impact of various types of culling for bTB control on badger social structure and movement [44, 96, 97]. Genetic tools based on non-invasive sampling have been used in Ireland and elsewhere to estimate badger group sizes at local scales [98, 99]. Kostka [98] estimated group size across all land classes in NI as 6.0 (s.e. 0.96, *n* = 22), greater than previous group sizes estimated by conventional methods, reviewed by Byrne et al. [15], but similar to studies undertaken in Britain (Judge et al. [99]; 6.74 (s.e. 0.63, *n* = 122)). Both studies indicated considerable variation in badger group size with a strong effect of landscape. Generally, in Ireland, however, there has been limited exploitation of non-invasive sampling, integrating genetic profiling, and statistical estimates of true population size, as well as elucidating population structure and relatedness. Furthermore, these types of approaches have not been developed at scale to help inform both local and national dynamics in a representative and robust design. Genetic tools also allow control of potential sources of pseudo-replication in a collection of (hair) samples that may be comprised of several individuals [19].

## 3. Parasites and Non-bTB Infections

Wildlife host species management can benefit from gaining knowledge of other factors affecting population dynamics, including parasite exposure. Furthermore, there is evidence which suggests that parasite coinfection dynamics can impact both the transmission and virulence of bTB infections [100].

### 3.1. Ecto- and Endo-Parasitology

Greater insights into the parasite communities of the badger have recently been made through surveillance activities associated with the bTB programme [30, 32]. These studies revealed a nematode-dominated helminth community in badgers. The composition was predominated by badger specialist species, *Perostongylus falciformis*, family Mustelidae specialists, *Uncinaria criniformis* and *Crenosoma melesi*, and a mammal specialist, *Eucoleus aerophilus* [101, 102]. Byrne et al. [102] also found badgers in Ireland to be infected with *Strongyloides* spp., two distinct unidentifiable nematodes and an unidentifiable larva. The lack of any cestode and trematode species and a reduced complement of nematode species infecting badgers in Ireland compared to GB and continental Europe could be due to the different ecology of badgers in Ireland. However, it is also likely that parasite diversity generally on the island is affected by island biogeography [103] and “host diversity-begets-parasite diversity” [104]. Protozoan parasites such as *Neospora caninum* and *Cryptosporidium* spp., which infect badgers elsewhere, have also not been detected in Ireland, although protozoan parasites infecting Irish badgers need further study [101]. Ectoparasites of badgers include the flea *Paraseras melis melis* and the mite *Trichodectus melis*, which are commonly found on badgers, their principal host [105]. The tick species include *Ixodes ricinus*, *I. canisuga*, and *I. hexagonus* [106]. *I. canisuga* is not known to transmit any significant pathogens, and the status of *I. hexagonus* as a disease vector in Ireland is not known [106, 107]. *I. ricinus* is the principal tick-borne disease vector in Ireland [107], but badgers have not been associated with this species. There has been little attention given to the impact of parasites on badger population dynamics in Ireland or the effect of badger management on parasite dynamics. However, a recent study has found associations between the prevalence of gut helminths and badger bTB status, suggesting that parasites could play a role in the epidemiology of bTB infection in wildlife [108]. One explanation for the association between helminths and bTB coinfection relates to the immunological impacts of parasitic infections eliciting different immunological pathways (Th1 vs. Th2), resulting in antagonistic effects.

### 3.2. Other Bacteria, Viruses, and Unknown Unknowns

Viral infections of badgers have not been well researched, with only a few in-depth studies in particular populations across the species range (see [109]). One exception is the recently described Mustelid gammaherpesvirus 1 (MusGHV-1), a sexually transmissible infection (STI) pathogen that is so far only found in badgers [110], where several cross-sectional and ecological studies have been undertaken in the United Kingdom (e.g., [111]). Reactivation of latent herpesvirus infection in the genitalia is known to cause localised clinical signs and may lead to reproductive failure in domestic animals. Tsai et al. [112] found that 50% (*n* = 102) of badgers culled in IE in April 2019 and February 2020 displayed macroscopic lesions that resembled lymphoid hyperplasia on the epithelium of the lower genital tracts. Histopathological examination of seven samples (five females and two males) concluded inflammation, with most exhibiting epithelial hyperplasia, and one (male) had intranuclear inclusion bodies in the epithelium, likely indicating viral infection. Genital swabs collected from 144 (71 males, 73 females) badgers tested positive by PCR for MusGHV-1. Tsai et al. [112] found that MusGHV-1 reactivation had a negative effect on female pregnancy rate but not on male fertility (sperm abundance or testes weight). Furthermore, analysis of adult badgers sampled in winter (*n* = 80) showed sex and age were risk factors for MusGHV-1 reactivation. The risk of MusGHV-1 reactivation was higher in males, and badgers, which were between 3 and 5 years old, had significantly lower reactivation rates than younger (2–3-year-old) ones. No MusGHV-1 was detected in foetal tissues from positive mothers (*n* = 5), suggesting that cross-placental transmission was unlikely. STIs that impact reproductive fitness have wide implications for badgers and for wild carnivores generally and may impact in circumstances of coinfection with bTB. Bovine TB in badgers can be associated with elevated cortisol levels and poor body condition, which have also been identified as stressors for MusGHV-1.

Given the experience of the COVID-19 pandemic, there is a global demand to invest in additional resources to explore the “unknowns” of wildlife diseases [113] and other zoonotic pathogens (e.g., antimicrobial resistant (AMR) pathogens [114] or other common zoonotic or livestock-associated pathogens, e.g., salmonellae [115]). This would promote an understanding of the diversity of pathogens that may be circulating within wildlife populations, including badgers in Ireland. Such knowledge would mean society was better equipped to respond to future epidemics that threaten vulnerable wildlife populations (conservation risk), domestic animals, and humans (One Health risk).

## 4. Badgers and Bovine Tuberculosis

### 4.1. M. *Bovis* in Badgers and Its Relationship with Cattle bTB

A role for the badger in bTB epidemiology was first proposed in the mid-20th century, when continental European researchers recorded badgers harbouring infection with *M. bovis* [116]. Thereafter, infected badgers were reported in GB [117], IE [118], and NI [119]. While there is some debate about the role badgers play in disease epidemiology in continental Europe (France and Spain), considerable evidence from Britain and Ireland suggests their involvement in the transmission of infection to cattle is significant [10]. Lines of evidence include culling intervention trials, prevalence (cross-sectional) studies, longitudinal studies, risk factor analyses, and molecular and whole genome sequencing epidemiological tracking.

Two large-scale badger culling trials were undertaken in IE in the late 1980s–1990s, both of which found significant reductions in cattle bTB risk associated with badger culling [13, 120]. Following these field studies, the primary approach to managing *M. bovis* in badger populations in IE (2004–2019) was targeted proactive culling in areas where cattle herd bTB “breakdowns” occurred [121]. These culled areas coalesced over time into larger management areas constituting up to 30% of the agricultural land area in IE [23]. Repeated culling of local populations resulted in significant declines in badger abundance based on capture metrics and indicators of badger presence [23, 122]. For example, the number of active openings at setts declined by 41%–82% in areas where culling occurred over a 6-year period. Therefore, the badger population density of IE has been depressed in areas of productive land for over a decade.

While accurate figures on the prevalence of *M. bovis* in badgers are recognised as being difficult to assess, given likely regional variation [123] and lack of sensitivity of diagnostic investigation tools [124], multiple surveys in recent years have indicated that a substantial proportion (12%–43% depending on the protocols used) of the Irish badger population is infected with *M. bovis* [123, 125]. Spatiotemporal analysis of the apparent prevalence of bTB in badgers culled (*n* = 4948) during the bTB eradication programme in IE (2007–2013) suggested that badgers were significantly more likely to test positive for bTB if they were in closer proximity to other infected setts (intraspecific infection clustering), and if they were male or a parous female relative to a female which had not conceived [123]. Furthermore, there was a significant positive association between local bTB infection levels in cattle and badger positivity risk at a scale of 1 km around badger setts [123].

Risk factor analyses have been undertaken in both IE and NI, associating the risk of bTB breakdown in cattle herds with metrics of badger abundance [126–128]. Across studies, there were positive associations between breakdown risk and metrics of badger abundance [126–128]. In NI, however, the risk of new breakdowns was highest in areas with high social group density and high levels of persecution [127]. Whether persecution was the cause (e.g., disturbing badgers, increasing infectious contacts) or a consequence (e.g., farmers attempting to reduce badger numbers under the assumption of lowering risk) of a bTB breakdown remains uncertain. In IE, follow-up analysis of herd breakdowns in areas that historically were part of a large-scale cull trial (four area trials [13]) revealed that measurable positive culling effects in cattle herds were still apparent 5–10 years after the trial [126]. Furthermore, the association between badger carrying capacity (potential density) and bTB risk waned over time as continued targeted culling reduced densities. In three of the study areas, there were trends towards decreasing risk with increasing culling efforts on targeted farms; however, this trend was reversed in a fourth area (Co. Donegal) [126]. Milne et al. [128] found positive univariable associations between bTB cattle herd breakdown duration and badger sett density in NI, but this association was affected by the inclusion of other spatial risk factors. The authors could not eliminate badgers as a source of infection, prolonging breakdown duration, due to confounding factors.

Molecular epidemiological studies in both IE and NI indicate that cattle and badgers in proximity share genetically similar strains of *M. bovis*, which is also consistent with past interspecific transmission of bTB [129, 130]. In NI, this relationship was observed across a wide geographic range. Whole genome sequencing (WGS) of *M. bovis* isolated from badgers and cattle is starting to reveal higher resolution phylogenetic data to infer more contemporaneous epidemiological links between cases and is helping to characterise transmission dynamics [131–134]. These applications to badger and cattle populations have been primarily conducted in NI, where ongoing interspecies transmission at the farm and patch levels have been demonstrated [131, 134]. Early indications from one study area are that intraspecies transmissions (cattle-to-cattle; badger-to-badger) predominate [134]. This pattern has also been seen in studies in GB [135–137]. One model from GB suggests that badger-to-cattle transmission is comparatively rare, accounting for ∼5% of introductions into cattle herds, but the impact of interspecific infections may be amplified by onward cattle-to-cattle transmission, indirectly affecting∼50% of herds [138]. Such amplification effects may not be readily detected by phylogenetic methods owing to the low mutation rate of *M. bovis* [83].

Statistical and mathematical modelling tools are essential for inferring the transmission dynamics of infection within badger populations and between badgers and cattle [67, 139–142]. Abdou et al. [143] paved the way regarding agent-based modelling of bTB dynamics *in silico* to inform the Irish bTB programme. This simulation model was used to estimate the impacts of various culling, vaccination, and combinatorial approaches (e.g., TVR) on a badger population across space and time, demonstrating the risk to population viability of long-term culling operations where immigration was limited. Mathematical modelling has been used to develop a two-host metapopulation model for bTB in NI [144], informed by advanced modelling of both the cattle and badger populations of NI [145, 146]. The other two host models from IE suggest that maintenance of infection within individual host populations, based on the estimated *R*_0_ (whereby infection is maintained where the effective *R*_0_ > 1, allowing for each primary case to infect, on average, more than one secondary case, or eradication where *R*_0_ < 1), can be reduced below 1, which could lead to disease eradication [147–149]. However, the relatively modest transmission of infection between hosts (cattle-to-badger and badger-to-cattle) appears to have resulted in maintenance at the system level (i.e., the *R*_0 system_ > 1), requiring additional management efforts to reduce interspecific transmission.

### 4.2. The Advent of Badger Vaccination as a Policy

While repeated culling of badger populations in areas of IE with endemic disease has reduced the risk of transmission of infection to cattle herds [13, 120], the policy is controversial, and the long-term prospects for culling as the only means of controlling infection have been deemed non-sustainable [126, 129, 150]. Data from GB, typically at higher density populations than found in rural Ireland, has suggested that badger culling is not always effective and could lead to increasing rates of spread of infection due to social disruption, increasing infectious contact rates, or stress-related immunological impacts. This is known as the “perturbation” hypothesis [73]. Other wildlife management options for disease control are limited [109]. However, vaccination was considered part of an “endgame” strategy for bTB control in IE [121, 150], subject to a consistent evidential base establishing that vaccination was technically and logistically feasible, cost-effective, and efficacious in terms of reducing transmission within badger populations and, by extension, between badgers and cattle [148, 151].

**Figure 1:**
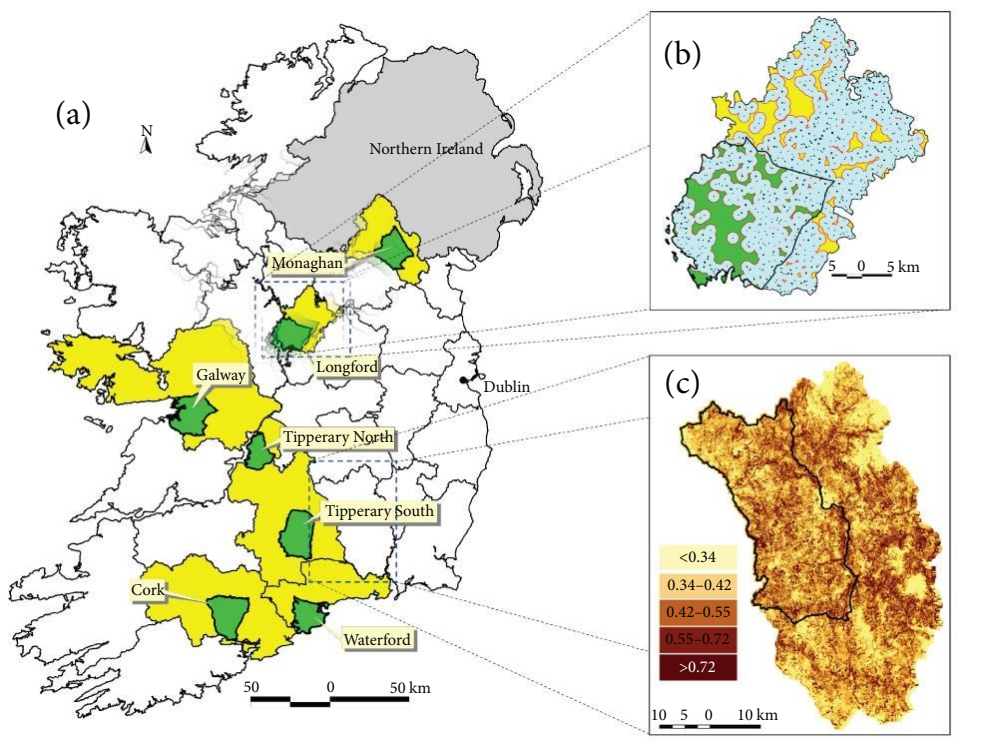
(a) Location of non-inferiority badger vaccine field study sites where vaccination areas (green) were compared with continued targeted culling (yellow) within the same county. (b) Details of one study area (Longford). Dots represent known sett (burrow) locations at the commencement of the study (*n* = 810; main = 193, non-main = 617); blue areas represent a 1 km buffered treatment area around each sett. Untreated areas within the vaccine area predominantly represent poor badger habitats. (c) County Kilkenny, with the location of the badger vaccination trial area depicted in black outline. The surface represents the relative probability of occurrence of badger groups.

**Figure 2:**
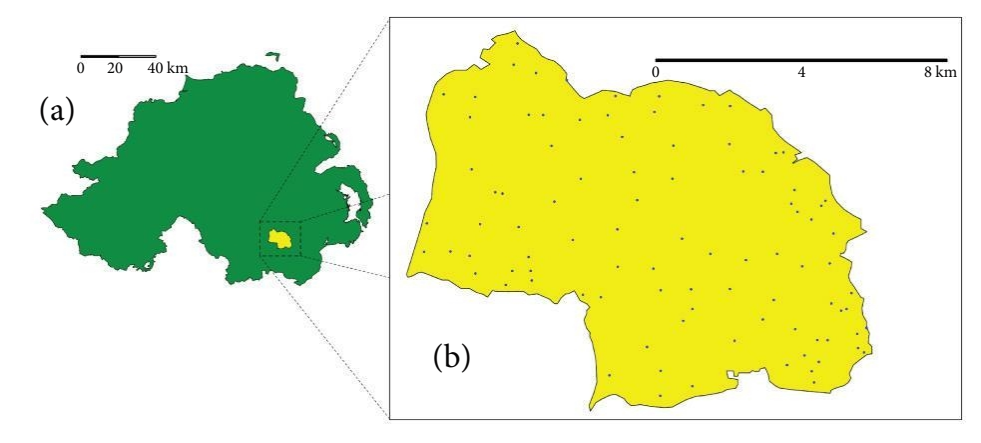
Map of Northern Ireland (a) depicting the location within the TVR intervention area and (b) including the location of badger setts within the study area.

Several laboratory studies have shown that vaccination can have benefits for reducing disease progression and severity in badgers [152]. However, extrapolation of any vaccine effects to a wild, non-controlled environment requires field-based studies [153]. Three large-scale vaccination-related field studies—the Kilkenny, IE, badger vaccination trial [148, 153, 154], the TVR field study in Co. Down, NI [26], and the non-inferiority wildlife intervention study in IE [24]—have been completed during the last decade in Ireland (Figures 1(a)–1(c) and 2(a), 2(b)).

The Kilkenny vaccination trial was designed to estimate the efficacy of Bacille Calmette Guerin (BCG) oral vaccination against naturally transmitted infection of *M. bovis* in badgers across a landscape of 755 km^2^ ([153]; Figure 1(c)). The landscape was divided into three intervention areas, where captured badgers were blindly 100% vaccinated orally or 50% vaccinated at random or 100% given a placebo dose. A transmission modelling approach was applied to the results of live sampled badgers using a serological test (Enfer chemiluminescent multiplex ELISA system) to measure seroconversion rates in vaccinated and non-vaccinated badgers which were infected during the trial. This suggested a vaccine efficacy for susceptibility, i.e., the ability to protect against infection following exposure, of 59% (95% CI = 6.5%–82%). There was no evidence for a benefit of vaccination for infectivity, i.e., reducing the risk of onward transmission from infected animals [148]. A separate analysis of data from the same trial using a different serological test (StatPak) and modelling approach demonstrated significant differences in time to seroconversion of infected badgers, depending on the vaccination status [154]. This gave rise to mean vaccine efficacy estimates between 36% (95% CI: −62%–75%) and 84% (95% CI: 29%–97%), depending on when badgers were enrolled into the trial. Follow-up research also showed the potential indirect benefits of vaccination at the population level, as evidenced by the lack of infection found in non-vaccinated badgers captured in areas that were targeted for 100% vaccination [155]. The model by Aznar et al. [148] allowed estimation of the *R*_0_ depending on vaccination coverage and suggested that 100% coverage would bring the reproduction ratio to 0.5. However, vaccination could still yield benefits (*R*_0_ < 1) at coverage levels of > 30%.

The TVR project aimed to test the feasibility of employing a “middle-ground” policy between culling and vaccination strategies, where only test-positive animals were culled and the remainder vaccinated, marked, and released ([26, 156]; Figure 2). Due to low positivity rates overall, the intervention resulted in only low levels of badger removals (4.1%–16.4% annually [26]) but correlated with a significant decline in apparent prevalence as the intervention advanced over the 5-year study. A Bayesian model, incorporating information from three diagnostic tests, estimated that the infection prevalence significantly declined (exponential model) across the years of the study, from 14% (95% credible interval (Crl): 0.10–0.20) to 1.9% (95% CrI: 0.8–3.8), with an annual reduction of 39.1% (95% CrI: 26.5–50.9) [156].

The non-inferiority wildlife intervention study was established to assess the relative performance of badger vaccination, based on capture and intramuscular vaccination with BCG, in comparison with continued culling as set out as part of the national policy. This was achieved by measuring cattle herd breakdowns in seven-paired sites across IE [24]. The hypothesis tested was that the outcome of vaccination of badgers was not inferior to continued badger culling (in impacting bTB in cattle). Across the seven sites, totalling 18,409 km^2^, vaccination was deemed non-inferior to culling in four sites. One site (Cork) provided ambiguous results. Vaccination was clearly inferior to the culling programme in another site (Monaghan), which had a high infection pressure. The final site (Galway), following post hoc analysis, was found to have significant differences in cattle populations between the vaccination and culled areas, confounding the treatment effect.

The results of the controlled experimental studies, field trials, and intervention studies informed the decision in 2019 in IE to move to incorporate badger vaccination into the bTB control programme, which has progressively expanded (Figure 1; [24]). In NI, there are no policies enacted involving wildlife vaccination at present, although public consultations and debates are ongoing at the time of writing (also see wildlife section below).

Vaccination programmes require knowledge of important ecological inputs on the changing dynamics of population structure over time to ensure appropriate levels of vaccine coverage are achieved. One of the difficulties is the development of a reliable test for bTB in badgers. Current tests for both cattle and badgers are time-consuming and costly. Some effort has gone into finding suitable alternatives using novel approaches, such as immunomagnetic separation [157] and thermal imaging [158].

## 5. Badger Management

**Figure 3:**
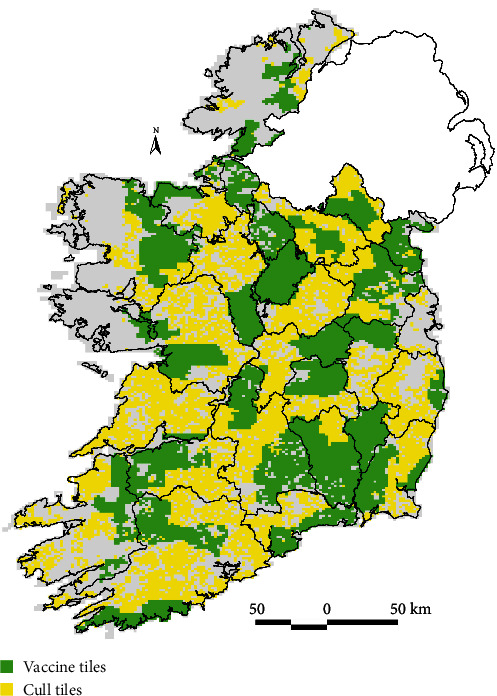
Distribution of areas where the badger management approach is either vaccination (green) or cull (yellow) up to March 2023, based on the management unit grid “tiles” of 2 ×3 km.

**Figure 4:**
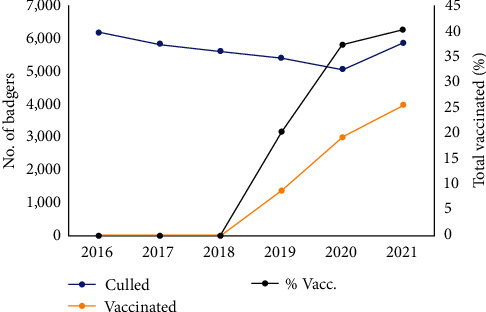
The number of individual badgers newly vaccinated, removed, and the proportion of total vaccinated per annum in IE from 2017 to 2021. Note more badger capturing for vaccination occurred during 2020–2021, but a proportion of badgers were recaptured/previously vaccinated.

Badgers have been culled as part of the IE bTB eradication programme since 2004, with the programme intending from the outset to reduce the national badger population by 20%–25% [150]. This was based on previous culling field studies undertaken since the late 1980s [13, 120, 159]. However, the strategy was considered a *medium-term* action, with the longer-term intention of managing the disease in wildlife with a vaccination strategy [150]. This was subject to the results of a significant body of fundamental and applied research on badger vaccination that has occurred in the interim period [56]. Furthermore, there were conservation safeguards included in the design of the strategy (e.g., limiting the land area that could be under capture), to mitigate the potential dangers to population viability. In IE, this included restricting capturing to agricultural land and no more than 30% of land area under population management. Since 2016, 5,000–6,000 badgers have been culled per annum in response to bTB breakdowns in cattle herds in IE (Figure 2), following a longer-term trend since 2004 (see gov.ie—Wildlife and TB (https://www.gov.ie); see Figure 3 for a map of current effort). However, in 2018, a new policy of badger BCG vaccination was launched based on the results of the Kilkenny vaccine trial and non-inferiority intervention study (Figure 1). Badger vaccinations increased during 2019–2021 (Figure 4). In 2021, 6,586 badgers were captured in vaccination areas, of which 3,958 were newly vaccinated (as badgers were not revaccinated if recaptured) (see [160]). The opposing impacts of these parallel policies (culling decreasing population density; vaccination increasing density due to alleviating the cull pressure, but also through reduced disease-induced mortality [143]) will mean badger populations are likely to experience different growth trajectories across space and time.

In NI, no badger culling interventions are currently part of the bTB eradication programme. However, the results of the TVR project produced “valuable data on the logistics and resources required to undertake a TVR approach to control *M. bovis* in badgers” [26], if policy changes. DAERA has recently called for an expression of interest for culling operations from not-for-profit companies [161], as part of a broader bTB eradication strategy [162]. Such proposed policies would include the controlled shooting of free-roaming badgers between dusk and dawn, supplemented by cage trapping and shooting, in areas of ≥100 km^2^. This proposed programme, if enacted, would emulate a similar policy employed in England since 2013, which has been associated with significant decreases in cattle herd breakdown risk in two of three monitored sites [163, 164].

Badger management attracts controversy on the island of Ireland, with both stakeholder opposition from conservation groups and support from farming interests. Furthermore, badger persecution in both IE (Irish Wildlife Act (1976)) and NI (Wildlife (NI) Order (1985)) is illegal, but disturbance and illegal culling continue to be recorded [165]. This persecution appears, at least in NI, to be more common where bTB levels in cattle are highest, possibly indicating “responsive persecution of badgers in high-cattle risk areas” [127]. Reid et al. [20] did not confirm the results of an earlier study [166] suggesting that sett disturbance negatively impacted the number of social groups and group size, as there was a significant decline in sett disturbance in NI between surveys in the early 1990s and 2007/8 but no change in badger abundance. Other drivers of badger mortality include road traffic accidents (RTAs) and bTB itself, although it appears to have only a small mortality effect [167] and may be ameliorated by vaccination [155]. Estimates of annual RTA mortality in IE and NI vary widely from 1.1% to 15% [10, 49, 168, 169]. This variation may reflect the true variation in badger density and the variation in road type (regional roads vs. dual carriageway vs. motorway) [63]. Despite the badger classified as “least concern” in the most recent Red List of mammals in Ireland [170], combinatorial effects of management and other pressures coupled with climate change [25] could have detrimental impacts locally.

## 6. Gap Analysis and Future Research

We have highlighted significant advances in research on badgers on the island of Ireland, which reflect broader research aimed at understanding wildlife populations and their management from conservation and One-health perspectives. Here, we identify gaps in our knowledge and potential routes by which these can be addressed (see Table S1 for a table of gaps).

As highlighted, estimating badger populations at local and national scales is an essential task in the future from both conservation and eco-epidemiological perspectives and will be essential going forward as different policy combinations of culling and vaccination may occur across the island. Potentially, the positive feedback between modelling (e.g., agent-based modelling [143]) and field data will be an efficient and cost-effective approach to managing the twin goals of maintaining population integrity (viability) with epidemiological goals (density thresholds, vaccine coverage, effort, etc.). Future proposed hierarchical geospatial analysis of datasets already available is currently in development (S. Ciuti, V. Morera-Pujol, pers. comm.), which should also help directly with optimising future prospective data collection. Furthermore, the use of alternative technologies and models for badger abundance is required (genetics, camera traps, remote sensing). Some techniques are already well developed (e.g., random encounter models [171, 172]), but hitherto have not been applied to badgers in published studies in Ireland or elsewhere.

Work on badger genetics to date has been based on the genotyping of 14 microsatellite markers, which, while useful, have limited resolution compared to dense, genome-wide arrays of genetic markers. It may well be that with greatly improved data, superior inferences on resistance to disease, landscape effects, and evolutionary questions can be made, which may be hastened with the recent completion of the badger genome. Furthermore, the integration of non-invasive sampling of badger and spatial statistical models is an essential future research goal. Developing a wildlife management genetic toolbox is an essential research need in Ireland to help support decision-making and decoupling of wildlife interventions from population estimates. Such approaches are currently being researched (A. McDevitt, D. O'Meara, pers. comm.) and should provide a framework for independent data sources for wildlife management as well as eco-epidemiological model validation.

Greater effort to understand the variation in bTB transmission dynamics is essential to help target if or when interventions may be most effective. Geospatial modelling of interspecific transmission risk, including the development of *R* (reproductive ratio) maps, will be an important step [139, 140]. There is a need to further develop the capacity in simulation and agent-based modelling to allow for advanced planning for wildlife management *in silico*. Such modelling facilitates cost-effective explorations of control options and could help inform on the risk of disturbance activities on bTB spread, but also the impact of interventions of differing types on the viability of badger populations. It is also important to understand how many wildlife host species maintain *M. bovis* within the environment and how they interact with badgers. Further agent-based modelling endeavours are planned, with potential development for multi-host system dynamics, including, e.g., badgers, deer, and cattle (K.J. Murphy, pers. comm.).

Better characterisation of the genomic diversity of *M. bovis* in Ireland and the spatial distribution of isolates among badgers will likely improve and clarify our understanding of the role that badgers play in the transmission of *M. bovis* to cattle. It may be the case that such transmission dynamics vary by region, where local contexts of host density, ecology, and disease prevalence differ. Therefore, future all-island analyses of transmission dynamics inferred from whole genome sequencing and phylodynamics will be an essential future research goal, coupled with advanced machine learning and artificial intelligence (AI) technologies. This work could also be informative for future monitoring of vaccination efficacy over time as interventions mature. Genomics may also help to clarify the relative importance of direct and indirect transmission routes, as currently there does not appear to be a consensus on which is the more important in terms of transmission dynamics.

The role of other pathogens, including parasites, in the population dynamics of badgers is currently poorly understood. Furthermore, the diversity of pathogens that badgers may be exposed to is currently understudied in Ireland and elsewhere [109]. AMR in wildlife sentinel species may help explain the spatial variation in selective pressures across landscapes and could be integrated with a broader bTB-directed badger management approach.

## 7. Conclusion

Wildlife management for disease control is technically and politically challenging and requires high-quality data streams to provide an evidence base for both policy and intervention. This is particularly the case with the badger-cattle-bTB episystem in Ireland. The primary wildlife host is legally protected, an important native species within an already depauperate mammal fauna, and yet has been culled. Significant advances have been made in the last decade in relation to estimating fundamental ecological (distribution, variation in abundance, movement ecology, phylogeography, and landscape genetics, factors impacting population dynamics) and epidemiological (transmission of infection between host species, direct and indirect contact points, effect of vaccination interventions) parameters of wildlife management import. There are opportunities to further exploit established and emerging technologies (genetic tools, remote sensing tech, tracking tech, etc.) and novel statistical and analytical tools (simulation, agent-based models, machine learning/AI) to help managers and decision-makers. For example, WGS data of *M. bovis* integrated with advanced phylodynamic modelling and machine learning can illuminate areas where shared epidemics between badgers and cattle occur or alternatively rule out infection sources. Furthermore, advances in badger population genetics could gain insights into how interventions impact genome-wide heterozygosity and, in turn, the complex relationships between badger population structure and bTB transmission and dispersal. Such technical advances need to be integrated into a broader multidisciplinary research agenda, where (human) stakeholders are engaged to ameliorate conflicts and improve human-wildlife coexistence. Together, such interdisciplinary research will fit within the broader goal of One Health, which seeks to “balance and optimise the health of people, animals, and the environment.”

## Data Availability

All data are presented within the paper.

## Abbreviations

BCG: Bacillus Calmette–Guérin
bTB: Bovine tuberculosis
GB: The island of Great Britain
IE: Ireland, state (also described as the Republic of Ireland)
MNA: Minimum-number-alive
mtDNA: Mitochondrial DNA
MusGHV-1: Mustelid gammaherpesvirus 1
NI: Northern Ireland, a devolved region of the United Kingdom
RTAs: Road traffic accidents
TVR: Test-and-vaccinate-or-remove
UK: United Kingdom of Great Britain and Northern Ireland, state
WGS: Whole genome sequencing.

## Supplementary Materials

Supplementary Material Supplementary material includes Table S1 outlining a gap analysis of areas that would benefit from additional research to understand badger ecology and their contribution to the epidemiology of bovine tuberculosis in areas where the species is a wildlife host.
