# Supplementary material for: Badger Ecology, Bovine Tuberculosis, and Population Management: Lessons from the Island of Ireland
Source: Transbound Emerg Dis. 2024 Jan 16;2024:8875146. doi: 10.1155/2024/8875146 (PMC12016995; doi:10.1155/2024/8875146)
Supplement: Supplementary Material — includes Table S1 outlining a gap analysis of areas that would benefit from additional research to understand badger ecology and their contribution to the epidemiology of bovine tuberculosis in areas where the species is a wildlife host. [file 8875146.f1.pdf]

SUPPLEMENTARY MATERIAL

Table S1: Gap analysis of areas that would benefit from additional research to understand badger ecology and their contribution to the epidemiology of bovine tuberculosis in areas where the species is a wildlife host.

Parameter	Problem	Current state	Future state	Gap	Action
Population size	Estimating national scale population estimates with precision and accuracy	NI: No national estimate since 2012; IE: Several modelling studies published but estimates uncertain	Robust, standardised, replicatable means of estimating and monitoring national population	Moving from snapshot, to monitoring population at the national level; devising approaches that are robust, replicatable, cost effective	Build advanced models that can deal with different data sources across scales to maximise current data utility; prospective design surveys
Population size	Consistent, comparable, cost-effective estimates of population size, preferably using non-invasive techniques with applications for disease control and wildlife management	Several studies have used trap-catch and CMR with different model types and estimators to establish metrics of population size	Robust, standardised, replicatable means of estimating and monitoring local populations, especially in response to disease control measures	Need for research into several non-invasive techniques; cross comparison between technologies and study designs (e.g. camera traps, genetics)	Research non-invasive approaches; cross compare current and novel techniques; integrate non-invasive with CMR data; utilisation of state-of-the-art statistical tools.
Movement	Moving from descriptions of wildlife movement to mechanistic understanding	Several studies have used traditional (CMR) and modern (GPS, proximity loggers, accelerometers etc) techniques to describe badger movements in different populations	Integration of movement data across populations to explore the mechanisms underlying the movement of badgers within their socio-spatial structures.	Currently badger movement has been well described, but there remains some mechanistic explanation lacking (e.g., intrinsic vs extrinsic factors)	Meta analyses on pooled movement data; build mechanistic models parameterised from empirical data; test hypotheses within simulation
Parasites	Explore how parasites are impacting on population dynamics of badgers, including the potential interactions with bTB and the impact of badger management strategies on parasite dynamics	Descriptive studies of endo- ecto-parasite communities have been undertaken; one paper describes potential for coinfection dynamics between helminths and bTB	Measure the impact of parasites on badger population dynamics, and establish where coinfection has meaningful impact on population dynamics and transmission	There are currently no studies evaluating parasite impacts on wildlife host population dynamics or how badger management will impact parasite dynamics	Integrate parasitology and studies investigating coinfection with bTB into prospective population field studies of badgers
Molecular genetics	Building up representation of samples for genetics study of wildlife host	Studies have been undertaken using moderate samples sizes to explore landscape genetics and ancestral analysis	Increase spatial and temporal resolution of samples for genetic analysis; higher resolution typing/sequencing technology used	Additional samples from underrepresented populations; application of novel and emerging sequencing tools and application of bioinformatic pipelines	Biobanking of host samples; research into novel and emerging genetic sequencing and analytical tools.
bTB (*M. bovis*)	Epidemiological studies on the association between badger density, culling (history), and vaccination impacts on bTB risk in cattle	Several studies have found associations between metrics of badger exposure and bTB risk, although the relationships do not appear to be simple, and may be	Better understanding of how badger wildlife interventions impact on local bTB risk in cattle populations, and how	Predictions on what we might expect from differing interventions is currently limited; further research on the impact of vaccination of badgers on cattle herd risk will be required to assess how the	Case-control, cohort, and longitudinal epidemiological study designs to explore patterns of risk relative to wildlife exposure and intervention histories; epidemiological assessment of the

		impacted by local conditions and intervention histories.	bTB may be spilling back to badgers depending on local cattle conditions	policy is being implemented and its impact	impact of vaccination on cattle herd bTB risk.
bTB (*M. bovis*)	Diagnostics of bTB in wildlife	Post-mortem diagnostics of bTB in badgers has been extensively researched; several pen side or lab-based antemortem tests have been used/trialled	Improved diagnostics with both high sensitivity and specificity, that can be used in the field, with minimal invasive sampling on live animals.	The current available pen side tests have modest test characteristics	Ensure promising emerging technology in diagnosing infection within wildlife is suitably researched for Irish conditions, and robustly evaluated.
bTB (*M. bovis*)	Using WGS and phylodynamics at both local and national scales within a multi-host bTB population	Several local studies have utilised WGS to demonstrate links between local badger, deer and cattle populations	Building up sample biobanks, including the prospective designed collection of samples to maximise information gain in terms of understanding spread of infection across different host species	Transitioning from local research led explorations of integrating WGS and phylodynamic modelling to all island scale, coordinated approaches	All island collaborative projects with directed and planned prioritisation of sampling and sequencing in cattle and wildlife; building up of expertise and international collaboration
bTB (*M. bovis*)	Greater linkages between advanced mathematical and simulation approaches to real-world, real-time wildlife-cattle data collection	Mathematical models have been used to establish the efficacy of vaccination intervention; simulation model was used to estimate the potential impact of TVR, culling, vaccination and combinatorial policy options	Extend simulation model to incorporate parameters with data derived from local populations, and allowing for more autonomous mode frameworks (e.g. allowing agents to make decisions and remember past events).	Reducing the gap between empirical driven research and model-based research; building model sophisticated multihost models to act as decision support tools for decision makers.	Continue with research integrating vaccination programmes with spatially explicit models of transmission risk; further development and utilisation of simulation environments to explore scenarios and policy options
